# Impact of Rheumatoid Arthritis on Pregnancy Outcomes: Increased Risks of Abortion and Low Birth Weight in a Prospective Case-Control Study

**DOI:** 10.31138/mjr.120225.rai

**Published:** 2025-12-31

**Authors:** Samar Al Emadi, Eman Satti, Nawal Hadwan, Naela Elmallahi, Priyanka Cackamvalli, Neethu Kunjumon, Fiaz Shamsul Alam, Nabeel Abdulla, Salwa Abu Yaqoub

**Affiliations:** 1 Rheumatology Section, Department of Medicine, Hamad Medical Corporation, Doha, Qatar;; 2 Women’s Wellness and Research Centre, Hamad Medical Corporation, Doha, Qatar

**Keywords:** autoimmune diseases, rheumatoid arthritis, case-control studies, infant, newborn, pregnancy complications, autoimmune, pregnancy outcome

## Abstract

**Objective:**

Rheumatoid arthritis (RA) is a chronic autoimmune disease posing significant challenges for women of childbearing age. This study investigated the impact of RA on pregnancy outcomes.

**Methods:**

A prospective case-control study was conducted at Hamad General Hospital, Qatar, from January 2016 to December 2021. It involved pregnant women with a confirmed diagnosis of RA and healthy pregnant controls. Data were collected through patient interviews and electronic medical records.

**Results:**

A total of 327 participants were included: 101 women with RA and 226 healthy pregnant controls. The mean disease duration among RA patients was 6.23±4 years. Active disease, defined by a Clinical Disease Activity Index > 2.8, was observed in 30.7% of patients in the second trimester and 26.7% in the third. Women with RA had significantly higher rates of miscarriage (19.8% vs. 0.9%; P<0.001), preterm birth (24.8% vs. 14.2%; P<0.001), and caesarean delivery (31.7% vs. 14.6%; P < 0.001) compared to controls. Multivariate analyses showed that RA was significantly associated with increased risks of a composite adverse pregnancy outcome, including miscarriage, intrauterine foetal demise, or intrauterine growth restriction (coefficient, B=2.876; P<0.001). Additionally, RA was linked to a threefold increase in the likelihood of low birth weight (OR=3.088; P=0.023) but a lower risk of neonatal morbidity (OR=0.385; P=0.034).

**Conclusion:**

RA adversely affects pregnancy outcomes, including higher risks of miscarriage, low birth weight, and preterm birth. These findings underscore the need for specialised prenatal care and close monitoring of disease activity and treatment during pregnancy to optimise maternal and neonatal outcomes.

## INTRODUCTION

Rheumatoid arthritis (RA) is a chronic autoimmune disease primarily targeting the joints but also various organ systems. It affects approximately 0.5–1% of the global population^1^ and around 0.16% in the Middle East and North Africa (MENA) region, including Qatar.^2,3^ This inflammatory condition is more prevalent among women, raising significant concerns about its potential effects on pregnancy outcomes for those of childbearing age.

Pregnancy in women with RA presents unique challenges due to the potential interplay between disease activity, medication, and pregnancy outcomes.^4,5^ While nearly half of pregnant women experience low disease activity or remission by the third trimester,^6,7^ up to 20% may face worsening symptoms or moderate-to-high disease activity, necessitating therapeutic intervention.^7,8^ Existing evidence indicates that RA is associated with increased risks of adverse pregnancy outcomes, such as preeclampsia,^9,10^ spontaneous abortion, preterm delivery,^5,10–15^ caesarean delivery,^5,10,11,15,16^ and hypertensive disorders of pregnancy,^16^ particularly in the context of active disease or use of some medications during pregnancy.^17^ The literature also reports various adverse neonatal outcomes, including an increased likelihood of low birth weight and small-for-gestational-age babies.^5,10,13–16^ congenital abnormalities,^5^ prolonged birth hospitalisation,^11^ and the need for neonatal intensive care unit admission.^10^ However, interpretation of these associations requires caution, as many studies fail to account for other contributing factors that may influence pregnancy outcomes in both the general and RA populations.

Most existing studies are retrospective, introducing limitations such as recall biases and incomplete clinical data. Additionally, large-scale database and cohort studies often overlook or inadequately adjust for critical confounders, including parity, reproductive history, medication exposure, and quality of prenatal care—all of which affect maternal and foetal outcomes.^5,7,18^ Thus, careful consideration of these confounding factors is essential when evaluating the influence of RA on pregnancy.^5,15^

Despite the prevalence of RA in women of childbearing age and the potential for disease flare-ups, no dedicated guidelines currently exist for the prenatal care of this population,^7,12,19–21^ Routine obstetric care is typically applied, likely due to limited robust data informing tailored care strategies. This absence of specific guidance underscores the need for focused studies to identify risk profiles and inform evidence-based recommendations for managing women with RA during pregnancy.^19–21^

The present prospective case-control study was designed to address this gap and aimed to identify the independent impact of RA on pregnancy outcomes after adjustment for known confounders. To the best of our knowledge, this study is the first of its kind in Qatar and one of very few in the region, with only one comparable investigation reported from Saudi Arabia.^17^

## MATERIALS AND METHODS

### Study design & setting

A case-control prospective cohort study was conducted at Hamad General Hospital in Qatar, from January 2016 to December 2021.

### Ethical Approval

This study was conducted following the ethical standards of the Declaration of Helsinki. It was approved by the Institutional Review Board of the Medical Research Centre at Hamad Medical Corporation (Date of IRB approval 16/4/2017; ethical approval number: MRC#15024/15). All participants provided written informed consent for data collection and research use.

### Participants

#### Inclusion and exclusion criteria

The case group comprised pregnant women diagnosed with RA, meeting the 2010 American College of Rheumatology/European League Against Rheumatism (ACR/EULAR) classification criteria.^22^ All cases were managed through a specialised rheumatology-pregnancy clinic at the largest tertiary centre in Qatar. Each pregnancy was treated as a distinct event, enabling the inclusion of multiple pregnancies per participant during the study period, as the study focused on individual pregnancy outcomes.

The study included a control group of healthy pregnant women without chronic medical conditions. Women with comorbidities known to adversely affect pregnancy outcomes, such as chronic diabetes, hypertension, and renal disease, were excluded from both groups. Hypothyroidism was allowed in both groups due to its high prevalence and manageable course. Twin pregnancies were also excluded.

#### Sociodemographic and clinical information

Sociodemographic, clinical, medication, and laboratory data were obtained from electronic medical records and patient interviews during routine visits to rheumatology-pregnancy and antenatal care clinics. All participants were followed across three antenatal visits and once post-delivery.

Collected variables included age, nationality, smoking status (active, passive, former, or never), weight, height (for baseline BMI calculation in the first trimester), total pregnancy weight gain (in kg), and obstetric history (gravidity and parity, including previous pregnancy losses). Additional RA-specific data included disease duration, number of pregnancies following RA diagnosis, and serological markers such as rheumatoid factor (RF) and anti-cyclic citrullinated peptide (anti-CCP) antibodies. Disease was considered active when the Clinical Disease Activity Index (CDAI) exceeded 2.8.^23^

Medications used to treat RA during pregnancy included oral corticosteroids, conventional synthetic disease-modifying anti-rheumatic drugs (csDMARDs) like hydroxychloroquine and sulfasalazine, biologic synthetic DMARDs (bDMARDs), immunosuppressive agents like azathioprine, nonsteroidal anti-inflammatory drugs (NSAIDs), and any other prescribed therapies.

### Variables

#### Pregnancy outcomes

Pregnancy-related complications, including gestational diabetes, pregnancy-induced hypertension, infections, and preeclampsia, were documented. Mode and timing of delivery—natural vaginal delivery (NVD), assisted delivery, or caesarean section—were also recorded. Pregnancy loss (miscarriage and intrauterine foetal demise—IUFD) was noted. Miscarriage, also known as spontaneous abortion, refers to natural pregnancy loss before 20 weeks of gestation, whereas IUFD is the foetal death that occurs inside the uterus after 20 weeks of gestation.^24^

#### Foetal outcomes

Gestational age at delivery was documented, with pre-term birth defined as a gestation of less than 37 weeks. Birth weight was recorded, and weights below 2.5 kg were considered indicative of low birth weight. Intrauterine Growth Restriction (IUGR) was identified based on ultrasound findings. Congenital anomalies detected at birth were also documented.

### Statistical methods

Descriptive statistics were used to summarise participant characteristics, with categorical variables expressed as frequencies and percentages and continuous variables presented as means ± standard deviations (SD) or medians with interquartile ranges (IQR), depending on the distribution. Data normality was assessed using the Kolmogorov–Smirnov test and Q-Q plots.

The association between categorical variables (demographic, clinical, and laboratory characteristics and their distribution among women with RA and without RA) was examined using the chi-square test or Fisher’s exact test, as appropriate. Mean values of quantitative variables were compared between two groups using the Student’s t-test and among more than two groups using one-way analysis of variance (ANOVA). The Mann-Whitney and Kruskal-Wallis non-parametric tests were used when a distribution was non-normal or variances were non-homogeneous. When a significant overall difference was observed while comparing more than two groups, pairwise comparisons were conducted using a suitable post-hoc test, with adjustment for multiple testing. The results were presented with the associated 95% confidence interval. The association between pregnancy outcomes and medication use among patients with RA was also examined using the chi-square test or Fisher’s exact test, as appropriate. Logistic regression analyses were performed using a generalised estimating equations (GEE) model to account for linked cases. This approach controlled for confounding variables and generated crude and adjusted odds ratios (OR) with corresponding 95% confidence intervals (CI). The analyses examined associations between disease presence and the following pregnancy outcomes: miscarriage, moderate and very preterm birth, low birth weight, congenital abnormalities, neonatal morbidities, and NICU admission. All analyses were adjusted for BMI, smoking, age at pregnancy, and parity.

Statistical analyses were performed using Statistical Package for the Social Sciences (SPSS) version 28.0 (SPSS Inc., Chicago, IL), with p-values < 0.05 considered statistically significant.

## RESULTS

A total of 327 participants were included in this study: 101 cases (pregnant women with RA) and 226 controls (healthy pregnant women). The mean age at pregnancy was 30.94 ± 5.53 years. The majority of participants were from the South Asian region (30%), followed by the Gulf region (28.5%) and the North Africa and Levant region (approximately 15% each). Additional nationality-related data are available in **Table 1**.

**Table 1.** Sociodemographic (cases and controls), disease characteristics, and medication use among patients with RA (cases).

**Description of cases N=101**	**N (%)**	
**Anti-CCP**	**Positive**	77 (76.2)
**Rheumatoid Factor**	**Positive**	76 (75.2)
**Duration of RA**	**Mean (SD)**	6.23 (4.00)
**Total weight gain during pregnancy^1^**	**Mean (SD)**	8.85 (4.86)
**Number of pregnancies after RA^2^**	**1 (current pregnancy)**	42 (41.6)
	**2**	23 (22.8)
	**3**	18 (17.7)
	**4**	13 (12.9)
Medication use in cases N=101*		**N (%)**
**Steroids**		30 (29.7)
**Hydroxychloroquine**		74 (73.3)
**Sulfasalazine**		45 (44.6)
**Azathioprine**		3 (2.9)
**TNF blockers**		26 (25.7)
**NSAIDS**		5 (4.9)

**Disease activity/CDAI in cases N=101**			
		**2^nd^ Trimester**	**3^rd^ Trimester**
**Disease Activity N (%)^3,4^**	**Not active (≤2.8)**	35 (34.7)	40 (39.6)
	**Active (>2.8)**	31 (30.7)	27 (26.7)
**CDAI^5^**	**Mean (SD)**	5.96 (9.31)	5.91 (11.51)
	**Min-Max**	0–37	0–49

Anti-CCP: Antibody cyclic citrullinated peptide; CDAI: Clinical Disease Activity Index; N: Number; NSAIDS: Nonsteroidal anti-inflammatory drugs; RA: Rheumatoid Arthritis; SD: standard deviation.

1- Number of patients does not sum up to 101 because of 54 missing values;

2- Number of patients does not sum up to 101 because of 5 missing values;

3- Number of patients does not sum up to 101 because of missing data (n_2nd trimester_=35; n_3rd trimester_=34);

4- Disease was considered active if the CDAI > 2.8;

5- Number of patients included in this analysis does not sum up to 101 because of missing data (n_2nd trimester_=35; n_3rd trimester_=34).

* Information was collected at the end of pregnancy (third-trimester visit), providing a snapshot of any medication taken at any point during pregnancy.

Sociodemographic characteristics, disease features, and medication profiles of RA patients (cases) are presented in **Table 1**. Participants with RA had a mean disease duration of 6.23 ± 4.00 years, with 76.2% testing positive for anti-CCP and 75.2% for RF. Regarding pregnancies after RA diagnosis, 41.6% reported one pregnancy, 22.8% reported two, 17.7% reported three, and 12.9% reported four pregnancies. Hydroxychloroquine was the most prescribed drug (73.3%), followed by sulfasalazine (44.6%). Steroids were used by 29.7% of patients, while biological agents, specifically TNF inhibitors, were used by 25.7%. Azathioprine and NSAIDs were less commonly utilised (2.9% and 4.9%, respectively), with NSAID use restricted to the first trimester only. Moreover, at 6 months before pregnancy, patients with active disease were treated more significantly with biologics (60% vs 27.8%). During the first trimester of pregnancy, no significant differences were found for any medication. During the second and third trimesters, patients with active disease were more significantly treated with steroids, sulfasalazine, and hydroxychloroquine (p<0.05 for all). Further medication details are provided in **Table 2**.

**Table 2.** Medication use among patients with RA (cases).

	Disease Activity*						
	**6 months before pregnancy**			**1^st^ Trimester**		
	**Active**	**Not Active**	P value**	**Active**	**Not Active**	P value**
**Steroids**	5 (33.3)	10 (27.8)	.956	4 (33.3)	7 (21.2)	.425
**Sulfasalazine**	8 (53.3)	18 (50)	.429	8 (66.7)	14 (42.2)	.257
**Hydroxychloroquine**	11 (73.3)	26 (72.2)	1.000	12 (100)	23 (69.7)	.097
**Azathioprine**	0 (0)	1 (2.8)	1.000	0 (0)	1 (3)	1.000
**NSAIDS**	0(0)	3 (8.3)	.564	0(0)	3(9.1)	.400
**Biologics**	9(60)	10(27.8)	**.002**	5(41.7)	11(33.3)	.125
	**2^nd^ Trimester**				**3^rd^ Trimester**	
	**Active**	**Not Active**	P value**	**Active**	**Not Active**	P value**
**Steroids**	14 (50)	6 (16.2)	**.012**	13 (59.1)	10 (24.4)	**.003**
**Sulfasalazine**	19 (67.9)	17 (45.9)	**.003**	19 (86.4)	16 (39)	**<.001**
**Hydroxychloroquine**	25 (89.3)	29 (78.4)	**.007**	21 (95.5)	31 (75.6)	**.006**
**Azathioprine**	0(0)	2 (5.4)	.725	0(0)	2(4.9)	.764
**NSAIDS**	1 (3.6)	3 (8.1)	.626	0(0)	5 (12.2)	.023
**Biologics**	10(35.7)	7(18.9)	.328	7(31.8)	7(17.1)	.250

* Disease was considered active when the CDAI was greater than 2.8.

** The Chi-square test (or Fisher's exact when expected count is lower than 5) was used.

Assessment of disease activity revealed that 30.7% of patients had active disease in the second trimester and 26.7% in the third trimester, as indicated by CDAI levels > 2.8 (**Table 1**).

**Table 3** presents a comparative analysis of demographic and pregnancy-related characteristics between cases (N = 101) and controls (N = 226). Women with RA were significantly older at pregnancy than controls (33.24 ± 5.00 years vs. 30.01 ± 5.44 years, P < 0.001). BMI distribution also differed significantly between groups, with a higher proportion of cases falling into the normal weight category compared to controls (34.7% vs.17.3%) and a higher percentage of over-weight individuals among controls compared to cases (78.8% vs. 49.5%; P < 0.001). Smoking status varied significantly (P < 0.001), with a higher proportion of smokers among controls (16.8%) versus RA patients (2%), most of whom were passive smokers.

**Table 3.** Comparative assessment between cases and control (demographics, pregnancy complications, postpartum data, and pregnancy outcomes).

			**Case** (101)	**Control** (226)	
			**N (%)**	**N (%)**	**p-value**
**Demographics**					
Age at pregnancy (Mean (SD))			33.24(5.00)	30.01(5.44)	<0.001 ^a*^
Nationality		Asian	46 (45.5)	78 (34.5)	0.181 ^b^
		Middle East	54(53.5)	137 (60.6)	
		African	0 (0)	5 (2.2)	
		European	1 (1)	4 (1.8)	
		American	0(0)	2 (0.9)	
BMI		Normal Weight	35 (34.7)	39(17.3)	<0.001^*^
		Overweight	50 (49.5)	178 (78.8)	
		No information	16 (15.8)	9 (4.0)	
Smoking		Smoker	2 (2.0) ^c^	38(16.8) ^d^	<0.001 ^b*^
		Non-Smoker	86(85.1)	152(67.3)	
		No information	13 (12.9)	36(15.9)	
Gravidity/parity (including miscarriage)^3^		0	18 (17.8)	68 (30.1)	0.060 ^b^
		1	33 (32.7)	64 (28.3)	
		2	27 (26.7)	40 (17.7)	
		4	23 (22.8)	52 (23)	
**Pregnancy complications**					
Gestational diabetes			8 (7.9)	43 (19)	<0.001 ^b*^
Gestational hypertension			1 (1)	1 (0.4)	<0.001 ^b*^
Infections			4 (4.0)	9 (4.0)	1.000^b*^
Others			10 (9.9)	45 (19.9)	0.076 ^b^
**Postpartum data**					
Weeks of gestation (Mean (SD))			35.16 (9.73)	38.75 (2.14)	<0.001 ^a*^
Preterm			25 (24.8)	32 (14.2)	<0.001^*^
Mode of delivery	NVD		37 (36.6)	160 (70.8)	<0.001^*^
	Assisted delivery		14 (13.9)	17 (7.5)	<0.001^*^
	C-Section		32 (31.7)	33 (14.6)	<0.001^*^
Birth Weight (Kg) (Mean (SD))			2.96 (0.54)	3.24 (0.46)	<0.001 ^a*^
LBW (Kg)			11 (10.8)	12 (5.3)	0.099
Neonatal morbidities ^e^			8 (7.9)	50 (22.1)	<0.001^*^
Congenital abnormalities			2 (2)	7 (3.1)	0.705 ^b^
NICU admission			8 (7.9)	11 (4.9)	<0.001^*^
**Pregnancy Outcomes**					
Miscarriage			20 (19.8)	2 (0.9)	<0.001^*^
IUFD			0 (0)	2 (0.9)	0.477 ^b^
Reported IUGR			2 (2)	0 (0)	0.095 ^b^
Live Birth			80 (79.2)	221 (97.8)	<0.001 ^b *^

C-section: Caesarean section; BMI: Body Mass Index; IUFD: Intrauterine foetal demise; IUGR: Intrauterine growth restriction. LBW: Low Birth Weight; NICU: Neonatal intensive care unit; NVD: Natural vaginal delivery; SD: Standard Deviation.

* Significant P value <0.05.

a Independent T-test;

b Fisher exact test;

c Including 2 passive smokers and no active smoker;

d Including 34 passive smokers and 4 active smokers;

e Jaundice and infection were the most common morbidities.

In terms of pregnancy complications, gestational diabetes was significantly more prevalent among controls than cases (19.0% vs. 7.9%, P < 0.001). Conversely, gestational hypertension was more prevalent in the RA group (P < 0.001).

### Neonatal Outcomes

Postpartum data on childbirth and neonatal outcomes are summarised in **Table 3**. Infants born to RA patients had significantly lower mean birth weights than those born to controls (2.96 ± 0.54 kg vs. 3.24 ± 0.46 kg, P < 0.001). Mean gestational periods were also shorter among cases (35.16 ± 9.73 weeks vs. 38.75 ± 2.14 weeks, P < 0.001), with a significantly higher prevalence of preterm deliveries among cases (24.8 % vs. 14.2%; P < 0.001). Normal vaginal delivery (NVD) was significantly more frequent in the control group (70.8%) than cases (36.6%), while the number of caesarean sections (C-sections) and assisted deliveries was higher in the RA group (C-sections: 31.7% vs. 14.6%, P < 0.001; Assisted deliveries: 13.9% vs. 7.5%, P < 0.001). Finally, neonatal morbidities were significantly more prevalent among controls (P < 0.001), whereas the number of NICU admissions was significantly higher among cases (P < 0.001).

### Pregnancy outcomes

Pregnancy outcomes are also summarised in **Table 3**. Participants with RA exhibited significantly higher rates of miscarriage compared to controls (19.8% vs. 0.9%, P < 0.001) and lower rates of live births compared to controls (79.2% vs. 97.8%, P < 0.001). Other complications did not show significant differences between the two groups.

### Multivariate analysis

**Table 4** presents the multivariate analysis of the association between various pregnancy outcomes and the presence of RA, adjusted for BMI, smoking status, age at pregnancy, and parity. Results revealed significant associations between RA and four specific outcomes: adverse pregnancy outcomes (including miscarriage, IUFD, or IUGR), low birth weight, C-section, and neonatal morbidities. Participants with missing values for particular categorical variables were assigned to specific categories, allowing their inclusion in the model despite incomplete information.

**Table 4.** Association between pregnancy outcomes and the disease adjusted for BMI, smoking, number of parities, and age at pregnancy.

**Model 1: Generalised Estimating Equations**				
		**Beta**	**CI 95%**	**P value**
Adverse Pregnancy outcome (Miscarriage Or IUFD Or reported IUGR* )				
Smoking (Yes vs. No* )		0.290	[−1.947;2.526]	0.800
BMI (overweight vs. normal weight* )		0.853	[−0.337;2.044]	0.160
**Parity**	(1 vs.0* )	1.602	[0.007;3.198]	**0.049^1^**
	(2 vs. 0* )	1.535	[−1.175;3.245]	0.078
	(4 vs. 0* )	0.722	[−1.170;2.613]	0.455
**Age at pregnancy**		0.004	[−0.106;0.113]	0.954
Group (Case vs. Control* )		2.876	[1.586; 4.166]	**<0.001^1^**

1 P Value<0.05 significant;

* Reference group

**Table:** 

**Model 2: Multinomial logistic regression**				
		**OR**	**CI 95%**	**P value**
**Dependent variable: Preterm outcome (<37 weeks of gestation)**				
Smoking (Yes vs. No* )		0.671	[0.211;2.139]	0.500
BMI (overweight vs. normal weight* )		0.495	[0.239;1.023]	0.058
Group (Case vs. Control* )		1.546	[0.751;3.180]	0.237
**Age at pregnancy**		1.016	[0.948;1.088]	0.658
**Parity**	(1 vs. 0* )	1.584	[0.724;3.465]	0.249
	(2 vs. 0* )	0.644	[0.230;1.804]	0.402
	(4 vs. 0* )	0.949	[0.330;2.725]	0.922
**Dependent variable: Low birth outcome (< 2.5kg)**				
Smoking (Yes vs. No* )		0.936	[0.190;4.618]	0.935
BMI (overweight vs. normal weight* )		0.448	[0.168;1.197]	0.109
Group (Case vs. Control* )		3.088	[1.164;8.193]	**0.023^1^**
**Age at pregnancy**		1.062	[0.965;1.169]	0.215
**Parity**	(1 vs. 0* )	0.659	[0.216;2.007]	0.463
	(2 vs. 0* )	0.393	[0.096;1.612]	0.195
	(4 vs. 0* )	0.566	[0.134;2.385]	0.438
**Dependent variable: Congenital anomalies**				
Smoking (Yes vs. No* )		2.823	[0.468;17.046]	0.258
BMI (overweight vs. normal weight* )		1.457	[0.268;7.922]	0.663
Group (Case vs. Control* )		1.198	[0.201;7.133]	0.843
**Age at pregnancy**		0.969	[0.828;1.135]	0.699
**Parity**	(1 vs. 0* )	0.940	[0.172;5.092]	0.940
	(2 vs. 0* )	0.609	[0.044;6.212]	0.609
	(4 vs. 0* )	0.798	[0.080;7.003]	0.798
**Dependent variable: Neonatal morbidities**				
Smoking (Yes vs. No* )		1.217	[0.525;2.821]	0.646
BMI (overweight vs. normal weight* )		0.932	[0.425;2.044]	0.860
Group (Case vs. Control* )		0.385	[0.160;0.930]	**0.034^1^**
**Age at pregnancy**		0.972	[0.909;1.039]	0.406
**Parity**	(1 vs. 0* )	0.797	[0.367;1.731]	0.566
	(2 vs. 0* )	0.835	[0.329;2.120]	0.704
	(4 vs. 0* )	0.804	[0.302;2.139]	0.662
**Dependent variable: NICU admission**				
Smoking (Yes vs. No* )		0.971	[0.191;4.938]	0.971
BMI (overweight vs. normal weight* )		0.458	[0.148;1.421]	0.176
Group (Case vs. Control* )		1.963	[0.617;6.240]	0.253
**Age at pregnancy**		0.943	[0.842;1.056]	0.308
**Parity**	(1 vs. 0* )	0.701	[0.203;2.419]	0.574
	(2 vs. 0* )	0.748	[0.163;3.432]	0.709
	(4 vs. 0* )	1.219	[0.252;5.883]	0.806

1 P Value<0.05 significant;

* Reference group

CI: Confidence Interval; BMI: Body Mass Index; IUFD: Intrauterine foetal demise; IUGR: Intrauterine growth restriction.

Women diagnosed with RA had a significantly increased risk of adverse pregnancy outcomes (coefficient B = 2.876, P < 0.001). Furthermore, these women had a threefold higher likelihood of delivering low birth weight infants compared to healthy controls (OR = 3.088; P = 0.023) and a higher probability of C-section (OR=2.55; p=0.018). Conversely, RA patients had a significantly lower risk of neonatal morbidities (OR = 0.385, P = 0.034).

## DISCUSSION

Based on current literature, this work is the largest prospective case-control cohort study conducted in Qatar and the Arab world using real-world data to compare pregnancy and foetal outcomes between women with RA and healthy pregnant women.

Despite adjustment for confounding variables such as BMI, smoking status, age at pregnancy, and parity, the risk of specific adverse outcomes remained significantly higher among RA cases, even though this group had lower rates of high BMI and smoking exposure. These findings suggest that RA is an independent risk factor for adverse pregnancy outcomes.

A significant association was observed between RA and the occurrence of at least one adverse pregnancy outcome, including miscarriage, IUFD, and IUGR. Miscarriage was the most significantly reported adverse pregnancy outcome, affecting nearly 20% of RA patients, which aligns with previous reports indicating a rate of 17%.^25^ Similarly, data from the Medical Birth Registry of Norway (1999–2009) showed increased risks of early and late miscarriage in women with RA, with relative risks of 1.2 and 1.4, respectively.^26^ Interestingly, a recent Mendelian randomisation study using data from pooled genome-wide association studies (GWAS) provided further support for the causal relationship, showing that genetically predicted RA was associated with both pregnancy loss (OR = 1.13) and IUGR (OR = 1.08).^27^ Additionally, studies have shown that high disease activity during pregnancy and the presence of anti-CCP antibodies are significantly linked to spontaneous abortion.^17, 25, 28–31^ In the present cohort, nearly 76% tested positive for both anti-CCP and RF, with active disease recorded in 31% and 27% of cases during the second and third trimesters, respectively. One proposed hypothesis for the elevated miscarriage rate is the presence of inflammatory and immunological responses characteristic of autoimmune rheumatic diseases, which may impair embryo implantation and interfere with decidualisation, thereby affecting pregnancy viability.^25,32,33^

Regarding IUGR, the present study identified a rate of 2%, slightly lower than the figures reported in previous literature (around 2.8^28^ and 3.5%^29^). Evidence from earlier studies indicates that the risk of IUGR is nearly doubled among women with RA compared to the general population (OR =1.93).^34,35^ Despite the relatively lower incidence observed in the present cohort, the clinical significance of IUGR remains considerable. This condition reflects a multifactorial process involving physiological and pathological factors affecting foetal development and serves as a reliable predictor of pregnancy outcomes.^36^ It could be linked to increased foetal and neonatal mortality and morbidity, leading to immediate perinatal complications.^36^

The current analysis found that women with RA were three times more likely to deliver infants with low birth weight (LBW) compared to healthy women. This observation aligns with a consistent body of literature across diverse ethnic groups and periods, which has consistently highlighted the detrimental effects of RA on birth weight.^5,10,13–15^ Studies have shown that mothers with RA, particularly those with high disease activity, are at increased risk of delivering babies classified as LBW or small for gestational age (SGA).^4,5,37–39^ A large population-based cohort study demonstrated that differences in birth weight between infants born to mothers with RA and those born to healthy mothers persist even after adjusting for variables such as infant sex, maternal age, socioeconomic factors, and maternal comorbidities.^15^ Similarly, another study reported that disease activity remained significantly associated with LBW after controlling for variables, including maternal smoking status, age, education, parity, newborn sex, and the use of assisted reproductive technologies.^4,38^

Several factors could contribute to restricted foetal growth and SGA outcomes, including genetic factors^13^ and high levels of circulating cytokines in the blood of pregnant women with RA.^40^ Additionally, the use of certain medications during pregnancy, such as prednisolone, may also affect foetal outcome.^38–41^ In contrast, biologic DMARDs and hydroxychloroquine are generally considered safe and are permitted during pregnancy according to current guidelines.^21,42,43^ While both pharmacological treatment and disease activity are recognised as critical determinants of neonatal outcomes, the present study was unable to assess their independent effects, mainly due to missing data.

Finally, individuals with RA were found to have a significantly lower risk of neonatal morbidities compared to women without RA. This result may appear surprising, as most existing studies report poorer neonatal outcomes and increased morbidity risks associated with RA.^5,8,15,17,44^ However, this finding should be interpreted cautiously, as the “other” morbidities in this category primarily included non-complicated conditions such as jaundice and infections, which are rarely emphasised in existing literature. Further research is needed to confirm this observation and elucidate the underlying mechanisms. It is also possible that enhanced clinical management and more vigilant follow-up contributed to these outcomes among RA patients in this study.

## CLINICAL IMPLICATIONS AND FUTURE DIRECTIONS

A carefully planned pregnancy, combined with a preventative strategy, is crucial to reducing the risk of RA-related complications.^30,40,45^ Women with RA would benefit from a comprehensive approach that enhances surveillance and the early detection and prediction of pregnancy complications, including growth failure. Timely interventions such as determining the delivery timing can significantly improve perinatal outcomes.^46^ This approach involves frequent monitoring of biochemical and clinical disease parameters, such as disease activity, regular Doppler and ultrasound assessments, and tailored counselling. For instance, at the medical centre in Qatar, women with RA receive preconception counselling that addresses disease management and medication use during pregnancy. This proactive approach aims to mitigate potential risks and optimise pregnancy planning. At a broader level, rheumatology and gynaecology societies should promote research initiatives aimed at exploring and understanding the risk factors associated with rheumatic diseases such as RA, thereby enabling the development of more robust evidence-based recommendations for management before and during pregnancy.

## LIMITATIONS AND STRENGTHS

This study has several limitations that warrant consideration. First, missing data for some variables, particularly disease activity, may have influenced the outcomes. While disease activity is a critical factor, its measurement was inconsistent across the cohort, limiting the ability to perform more complex analyses and fully assess its impact, especially in relation to medication use. Similarly, the evaluation of IUGR relied on ultrasound reports, which may not provide completely accurate assessments.

Although the study adjusts for principal confounders, including BMI, smoking status, and parity, unmeasured factors such as socioeconomic status, dietary habits, sleep patterns, and genetic predisposition may contribute to the observed associations. The absence of these variables introduces potential residual confounding, which should be considered when interpreting the findings. Additionally, paternal influences—including genetic predisposition, health conditions, and socioeconomic factors—were not assessed. While these factors may affect pregnancy outcomes, their exclusion limits the ability to evaluate their impact. Future research incorporating these variables could provide a more comprehensive understanding of post-pregnancy complications.

A further limitation is that the study focused exclusively on immediate postpartum outcomes. Future research with extended follow-up is necessary to clarify the long-term neonatal consequences.

Despite these limitations, this study represents one of the first prospective case-control investigations conducted in Qatar and the Arab world using real-world data. It provides unique insights into the specific impact of RA on pregnancy outcomes within this regional context. Appropriate statistical methods were employed to account for missing data, and a logistic regression analysis was performed to adjust for confounding factors that may affect maternal and foetal outcomes. Consequently, the findings offer valuable contributions to the field, informing clinical practices and guiding future research directions in this population.

## CONCLUSION

The findings from this study highlight the association between rheumatoid arthritis and adverse pregnancy outcomes, including miscarriage, intrauterine growth, and low birth weight. These results reinforce the need for closely monitoring of women with RA before and throughout pregnancy to mitigate potential risks. Future research should further examine contributing factors, such as smoking status, body mass index, age at gestation, disease activity, pharmacological treatment, and relevant biological markers, among women with RA. An enhanced understanding of these elements would support clinicians in optimising follow-up care, identifying modifiable factors, and reducing the likelihood of obstetric and adverse foetal outcomes in this population.
